# Phosphorylated toll-like receptor 4 defines a high-risk sepsis endotype

**DOI:** 10.1186/s13054-026-06115-5

**Published:** 2026-05-30

**Authors:** Madita Mühlhaus, Birte Dyck, Andrea Witowski, Matthias Unterberg, Alexander Wolf, Helge Haberl, Alexander von Busch, Dominik Ziehe, Patrick Thon, Lars Palmowski, Britta Westhus, Stefan Felix Ehrentraut, Thilo Bracht, Malte Bayer, Barbara Sitek, Katrin Marcus, Martin Eisenacher, Björn Ellger, Frank Wappler, Elke Schwier, Dietrich Henzler, Thomas Köhler, Alexander Zarbock, Christian Putensen, Ulrich Hermann Frey, Moritz Anft, Nina Babel, Hartmuth Nowak, Tim Rahmel, Michael Adamzik, Katharina Rump, Björn Koos, the SepsisDataNet.NRW research group

**Affiliations:** 1 Klinik für Anästhesiologie, Intensivmedizin und Schmerztherapie, Ruhr-University Bochum, Knappschaft Kliniken Universitätsklinikum Bochum, Zentrum für perioperative Präzisionsmedizin, ZKF1, Universitätsstraße 150, Bochum, 44801 Germany; 2 Klinik für Anästhesiologie, Intensivmedizin und Schmerztherapie, Ruhr-University Bochum, Knappschaft Kliniken Universitätsklinikum Bochum, Intensivmedizin und Schmerztherapie, Bochum, Germany; 3 Klinik für Anästhesiologie und Operative Intensivmedizin, Universitätsklinikum Bonn, Bonn, Germany; 4 Medizinisches Proteom-Center, Ruhr-University Bochum, Bochum, Germany; 5 Medical Proteome Analysis, Center for Proteindiagnostics (PRODI), Ruhr University Bochum, Bochum, Germany; 6 CUBiMed.RUB, Medical Faculty, Ruhr University Bochum, Bochum, Germany; 7 Klinik für Anästhesiologie, Intensivmedizin und Schmerztherapie, Knappschaft Kliniken Dortmund, Dortmund, Germany; 8 Department of Anaesthesiology and Operative Intensive Care Medicine, University of Witten/Herdecke, Cologne Merheim Medical School, Cologne, Germany; 9 Department of Anesthesiology, Surgical Intensive Care, Emergency and Pain Medicine, Ruhr-University Bochum, Klinikum Herford, Herford, Germany; 10 Department of Anesthesiology and Intensive Care Medicine, AMEOS-Klinikum Halberstadt, Halberstadt, Germany; 11 Klinik für Anästhesiologie, Operative Intensivmedizin und Schmerztherapie, Universitätsklinikum Münster, Münster, Germany; 12 Marien Hospital Herne, Universitätsklinikum der Ruhr-Universität Bochum, Bochum, Germany; 13 Center for Translational Medicine, Medical Clinic I, Marien Hospital Herne, University Hospital of the Ruhr-University Bochum, Herne, Germany; 14 Klinik für Anästhesiologie, Intensivmedizin und Schmerztherapie, Ruhr-University Bochum, Knappschaft Kliniken Universitätsklinikum Bochum, Zentrum für Künstliche Intelligenz, Medizininformatik und Datenwissenschaften, Bochum, Deutschland; 15 Klinik für Anästhesiologie, Intensivmedizin und Schmerztherapie, Zentrum für klinische Proteomik und Metabolomik, Ruhr-University Bochum, Knappschaft Kliniken Universitätsklinikum Bochum, Bochum, Deutschland

**Keywords:** Sepsis, 30-day-survival, Proximity ligation assay, TLR4 receptor, TLR4 phosphorylation, SepsisDataNet.NRW, Endotype, Precision medicine

## Abstract

**Background:**

Sepsis is a life-threatening condition characterized by a dysregulated immune response to infection. Toll-like receptor 4 plays a central role in pathogen recognition and inflammatory signalling and has been considered a key driver of sepsis pathophysiology. Pharmacological inhibition of this receptor showed beneficial effects in experimental models but failed in clinical trials. We therefore aimed to quantify in vivo activation of Toll-like receptor 4 in patients with sepsis and to determine its association with 30-day survival.

**Methods:**

Peripheral blood mononuclear cells were obtained from 100 patients with sepsis enrolled in the SepsisDataNet.NRW cohort. Samples were collected on day 1 (within 36 h after diagnosis) and day 4. Activation of TLR4 was quantified by measuring receptor phosphorylation using a validated proximity ligation assay. Survival analyses were performed using Kaplan-Meier curves and Cox proportional hazards regression models to assess the association between receptor activation and 30-day mortality.

**Results:**

Overall activation of TLR4 was low, with median values below one signal per cell at both day 1 and day 4. Despite the generally low levels, a subgroup of patients showed increased receptor activation. Higher activation was associated with significantly reduced 30-day survival. Patients with elevated activation had a higher risk of death both at day 1 (HR 2.03, 95% CI 1.01–4.07, *p* = 0.048) and day 4 (HR 2.77, 95% CI 1.14–6.73, *p* = 0.025). This association remained significant after adjustment for SOFA score at admission, age, infection focus and sex in multivariable Cox regression analysis (*p* = 0.006).

**Conclusions:**

In vivo activation of TLR4 is not uniformly present in patients with sepsis but occurs only in a subset of individuals. In those patients, increased activation is strongly associated with mortality. These findings suggest the presence of a distinct high-risk sepsis endotype characterized by enhanced receptor activation. This may help explain the failure of previous clinical trials of TLR4 inhibitors and supports the concept of biomarker-guided precision medicine approaches in sepsis.

**Trial registration:**

German Clinical Trials Register (DRKS), DRKS00018871, retrospectively registered on 14 November 2019.

**Supplementary Information:**

The online version contains supplementary material available at 10.1186/s13054-026-06115-5.

## Background

Sepsis is a complex and life-threatening disease [1], claiming millions of lives each year [2]. It is defined as an acute organ dysfunction caused by a dysregulated host response to an infection [3]. Despite significant advances in the understanding of the underlying pathomechanisms, a causative treatment to decrease mortality, besides antibiotic treatment and source control, has yet to be found. This is due to the varied and often unpredictable clinical manifestations of sepsis, making the patient group highly heterogeneous [4, 5]. Hence, it is desirable to intervene as early as possible in the course of the disease. This pursuit has caused the focus of interest to shift to the first line of response in innate immunity, namely the pattern recognition receptors (PRRs), particularly the Toll-like receptor (TLR) family. TLRs are transmembrane glycoproteins and are responsible for detecting damage-associated-molecular-patterns (DAMPs) and pathogen-associated-molecular-patterns (PAMPs) [6]. Each TLR is activated by its own set of ligands. TLR4 is primarily known for its ability to recognize lipopolysaccharides (LPS), a component of the Gram-negative bacterial membrane. Notably, TLR4 activation requires phosphorylation of its own intracellular domain [7]. Upon activation, the receptor induces a primary pro-inflammatory immune response via the myeloid differentiation primary response 88 (MyD88)/nuclear factor-kappa B (NF-κB) axis [8]. This results in the production of cytokines such as tumor necrosis factor alpha (TNF-α) and interleukin-6 (IL-6) [9], as well as reactive oxygen species (ROS), acute phase proteins and antimicrobial peptides [10].

These findings suggest that TLR4 serves as a key pro-inflammatory stimulus in sepsis, contributing to various potentially life-threatening dysfunctions such as loss of total peripheral resistance, loss of blood pressure and cardiac depression [11, 12]. Therefore, inhibiting TLR4 could potentially prevent an excessive inflammatory response. On the one hand, this therapeutic strategy was indeed successfully tested in mouse models, showing a significant decrease in mortality when TLR4 was deleted or inhibited [13]. This might be due to the observed preservation of cardiac and vascular function and reduced cardiac hyperinflammation following TLR4 antagonism [14–16]. On the other hand, inhibiting or deleting TLR4 may increase susceptibility to non-severe Gram-negative infections, leading to increased mortality rates [13, 17]. In line with this discrepancy, large clinical trials such as the ACCESS study, which tested Eritoran, have failed to demonstrate positive survival effects of TLR4 inhibition [13, 18]. Moreover, it is noteworthy that the activation state of TLR4 has never been explicitly assessed in patient material, which means that clinical trials were conducted independently of TLR4 activation status. Thus far, quantifying TLR4 activity has mainly relied on downstream activation and cytokine measurement, such as NF-κB nuclear translocation or expression of IL-6. These were then inferred to reflect receptor activation, which is highly unspecific, as both molecules are not exclusively activated by the TLR4 network [19–25]. 

We recently established a proximity ligation assay (PLA) for the quantification of phosphorylated TLR4, which allows for a more precise evaluation and quantification of the receptor activation state [10]. 

Therefore, we tested the hypothesis that septic patients show a homogeneously elevated activation of TLR4 and that the activation status is associated with 30-day survival.

## Methods

### Patient recruitment

The samples used in this study were part of the multicentric SepsisDataNet.NRW study (German Clinical Trial Registry No. DRKS00018871), which prospectively enrolled patients fulfilling the Sepsis-3 criteria between March 1, 2018, and May 31, 2022. Patients were recruited and treated at intensive care units of seven university or tertiary care hospitals in the German state of North Rhine-Westphalia. This study was approved by the Ethics Committee of the Medical Faculty of Ruhr-University Bochum (Registration No. 18-6606 – BR and 22-7477) or the responsible ethics committee of each respective recruitment center.

Inclusion criteria comprised adult patients (≥ 18 years) with a diagnosis of sepsis according to Sepsis-3 criteria and availability of biomaterial at the defined study time points (day 1 and day 4). No specific exclusion criteria were applied regarding underlying malignancies or ongoing immunosuppressive therapies, in order to reflect a real-world ICU population.

Within the framework of SepsisDataNet.NRW, biomaterials including whole blood were collected at day 1 (within 36 h after sepsis diagnosis) and day 4 after study inclusion. These samples were utilized for serum, plasma and peripheral blood mononuclear cells (PBMCs) extraction. Patients who were lost to follow-up, or for whom data on SOFA score, focus of infection, or age could not be obtained were excluded.

Control patients were eligible if they were ≥ 18 years of age, underwent planned intermediate- to high-risk surgery with scheduled postoperative intensive care monitoring, provided written informed consent, and did not fulfil Sepsis-3 criteria for sepsis or septic shock at enrolment. Exclusion criteria were age < 18 years, emergency surgery, fulfilment of Sepsis-3 criteria for sepsis or septic shock, prior participation in SepsisDataNet.NRW or the control cohort, participation in another clinical study, or absence of informed consent. Biosamples from control patients were processed using the same protocols as sepsis samples.

### Sex as a biological variable

This study included both male and female patients. Sex was considered as a biological variable in all analyses. No significant differences in TLR4 phosphorylation levels or their association with 30-day survival were observed between male and female patients; therefore, data from both sexes were analyzed together.

### Microbiological classification and gram-negative pathogen detection

Microbiological classification was based on pathogen identification from blood cultures and, where available, clinically relevant non-blood microbiological specimens obtained at the time of sepsis diagnosis. Non-blood specimens were considered only if they were obtained within the diagnostic time window and corresponded to the documented infection context. Screening swabs, fungal isolates, and findings judged to represent colonization or contamination were not used for bacterial Gram classification. Because the biological question concerned potential exposure to LPS derived from Gram-negative bacteria, polymicrobial infections containing Gram-negative bacteria were classified as “Gram-negative pathogen detected” rather than as exclusively Gram-negative infections. Patients with bacterial pathogen detection but without Gram-negative bacteria were classified as “Gram-positive pathogen detected without Gram-negative detection”. Patients without relevant bacterial pathogen identification were not included in pathogen-based subgroup analyses.

### PBMC isolation and plasma collection

Peripheral blood mononuclear cells (PBMCs) were isolated from the blood of septic patients to assess Toll-like receptor 4 (TLR4) activation status via proximity ligation assay. Blood was collected in 9.0 mL EDTA monovettes (Sarstedt, Nümbrecht, Germany) and subsequently subjected to Ficoll density gradient centrifugation (GE Healthcare Europe GmbH, Freiburg, Germany). The plasma supernatant was collected and stored at − 80 °C for further proteomic analysis, and the PBMC phase was also collected, washed with phosphate-buffered saline (PBS), and residual erythrocytes were removed by lysis with water. The purified PBMCs from all patients and controls were subsequently cryopreserved using Bambanker freezing medium (Nippon Genetics, Düren, Germany) in liquid nitrogen at − 196 °C until use.

Prior to further analysis, cells were thawed, counted, and centrifuged onto microscope glass slides using a cytocentrifuge (Cellspin II, Tharmac GmbH, Wiesbaden, Germany). Cells were then fixed in 4% formaldehyde (Carl Roth GmbH + Co. KG, Karlsruhe, Germany) in PBS. Prepared slides were air-dried and stored at − 80 °C until further processing.

### Serum collection

For serum collection, peripheral venous blood was drawn into 9.0 mL Serum CAT tubes (Sarstedt, Nümbrecht, Germany) and left for at least 30 min. Subsequently the samples were centrifuged at 4,000 × g for 4 min. The serum supernatant was aliquoted and stored at − 80 °C until further analysis.

### Proximity ligation assay

The proximity ligation assay (PLA) was performed as described previously [10]. Briefly, the cells underwent rehydration and permeabilization using 0.1% Triton X (Carl Roth, Karlsruhe, Germany), followed by treatment with 1% SDS (Carl Roth, Karlsruhe, Germany). Non-specific binding sites were blocked using Duolink Block (Sigma-Aldrich, St. Louis, USA). The primary antibodies against TLR4 (sc-293072, Santa Cruz Biotechnology, Dallas, USA) and phospho-tyrosine (p-Tyr-1000, #8954, Cell Signaling Technology, Denver, USA) were diluted 1:50 in antibody diluent (Sigma-Aldrich, St. Louis, USA) applied to the slides, and then incubated at 4 °C, overnight. Similarly, the primary antibodies against TLR4 (AF1478, R&D Systems, Minneapolis, USA) and MyD88 (sc-74532, Santa Cruz Biotechnology, Dallas, USA) were diluted 1:100, and incubated on the slides overnight at 4 °C. The unbound primary antibodies were removed by washing the next day. The slides were then incubated with the proximity probes Duolink Mouse Plus and Duolink Rabbit Minus (Sigma-Aldrich, St. Louis, USA), according to the manufacturer’s instructions. Subsequent steps included ligation and amplification using a previously described protocol [10]. The slides were mounted in Slowfade Gold antifade medium, and nuclei were counterstained with DAPI. Finally, the slides were imaged using a wide-field microscope (IX51, Olympus, Tokyo, Japan).

The specificity of TLR4 phosphorylation detection was further validated by TLR4 siRNA-mediated knockdown followed by qRT-PCR and immunofluorescence analysis as well as single-antibody controls. Detailed methods and validation experiments are provided in the Supplementary Data (see Supplementary Table 1, Supplementary Figs. 1, 2 and 3).

### Image analysis

FIJI (Image J) and CellProfiler (Stirling, Swain-Bowden et al. 2021) were used for image analysis. To avoid incorrect selection of cellular debris, intact nuclei were selected and marked using the DAPI channel. In FIJI, colour contrast was optimized by calculating maximal intensity projections, and images compatible for CellProfiler were created.

Images were analysed in CellProfiler and subjected to a pipeline containing the following modules: Identify Primary Objects (nuclei), Identify Secondary Objects (cells), Enhance or Suppress Features, Identify Primary Objects (PLA signals), and Relate Objects (PLA signals to cells). For each sample approximately 100 cells were analyzed, with at least three images per sample. In rare cases, when 100 intact cells could not be identified a minimum of 30 cells were analysed.

### Measurement of cytokine serum concentration

The cytokine serum concentrations were measured on day 1 and day 4 after study inclusion. To quantify the cytokines the LEGENDPlex Human Inflammation Panel 1 (BioLegend, San Diego, USA) was utilized according to the manufacturer’s instructions. This panel includes the following cytokines: IL-1β, IL-6, IL-8, IL-10, IL-12p70, IL-17 A, IL-18, IL-23, IFN-α, IFN-γ, TNF-α, MCP-1 (CCL2), and IL-33.

Briefly, the antigens were captured by incubating the serum samples with LEGENDPlex beads, followed by washing steps and application of detection antibodies. The fluorescence was then quantified using a flow cytometer (Canto II, BD Biosciences, San Jose, USA). In cases where the recorded cytokine concentration fell below the lower limit of detection (LOD), a value of 0 pg/mL was assigned, whereas values exceeding the upper LOD were set to the upper LOD.

### Statistics

Statistical analysis was performed using SPSS (IBM, Armonk, New York USA, version 28) and GraphPad Prism (Ja Jolla, California, USA, Version 8). Data are presented as median [IQR] unless stated otherwise. Differences in TLR4 expression after knockdown were tested using an unpaired t-test or the Mann-Whitney U test as appropriate. Correlations between TLR4 activation and serum cytokine concentration in patients were calculated using Spearman correlation analysis. In addition, temporal consistency of TLR4 activation was assessed using Spearman’s rank correlation coefficient to evaluate the association between day 1 and day 4 values. Furthermore, patients were stratified based on day 1 TLR4 activation, and differences in day 4 activation between groups were analysed using the Mann–Whitney U test. Differences in TLR4 phosphorylation between patients with Gram-negative pathogen detection and patients with Gram-positive pathogen detection without Gram-negative detection were tested using the Mann–Whitney U test. Thresholds of the TLR4 signals per cell for the Kaplan-Meier survival analysis were determined by receiver operating characteristic (ROC) curve analysis. The area under the curve (AUC) with 95% confidence intervals and corresponding p-values were calculated. Optimal cut-offs were determined using Youden’s index. Based on these thresholds, sensitivity, specificity, positive predictive value (PPV), and negative predictive value (NPV) were calculated (see supplementary data for more detail). The log-rank test was used to determine the statistical significance of the observed survival effect. Hazard ratios were determined using univariate Cox regression analysis. We evaluated the independence of the TLR4 effect on survival from SOFA score, age, infection focus and sex using a multivariable Cox regression analysis.

### Study approval

This study was approved by the Ethics Committee of the Medical Faculty of Ruhr-University Bochum and the respective sites (Registration No. 18-6606 – BR for recruitment and 22-7477 for analysis). All patients provided informed consent. If patients were unresponsive, an independent physician was consulted and patients provided informed consent after their recovery according to the regulations of the Ethics Committee.

## Results

### Patient characterization

After verification of the assay, TLR4 activation was quantified in the PBMCs of 100 septic patients. At study inclusion, the median sequential organ failure assessment (SOFA) score of the cohort was 9 [IQR: 6–11]. The median age was 64 years [IQR: 52–74] and 64% of patients were male (Table 1). The most common focus of infection was the lower respiratory tract, which was found in 48 patients and intra-abdominal focus identified in 25 patients. 30-day mortality was 32% in the patient group.

**Table 1:** Baseline characteristics of the sepsis patient cohort

	Entire cohort	*n*
***n***	100	100
**Male gender n (%)**	64 (64)	100
**Age years median [IQR]**	64 [52–74]	100
**SOFA score median [IQR]**	9 [6–11]	100
**SAPS-2 DRG median [IQR]**	36 [28–44]	93
**PCT ng/mL median [IQR]**	2.6 [0.5–11.5]	84
**CRP mg/dL median [IQR]**	17 [11–28]	82
**Lactate mM median [IQR]**	1.4 [0.9–1.9]	84
**Comorbidities n (%)**	92	92
** Alcohol**	8 (9)	
** Chronic kidney disease**	25 (27)	
** Hypertension**	67 (73)	
** Diabetes**	26 (28)	
** Obesity**	24 (26)	
** Cardiovascular**	34 (37)	
** Malignancies**	22 (24)	
** Nicotine**	17 (19)	
** Dialysis**	7 (8)	
** Transplantation**	19 (21)	
** COPD**	14 (15)	
** Other (lungs)**	8 (9)	
**Focus of infection n (%)**	100	100
** Central nervous system**	4 (4)	
** Lower respiratory tract**	48 (48)	
** Skin and soft tissue**	4 (4)	
** Genitourinary**	7 (7)	
** Cardiovascular**	4 (4)	
** Intra-abdominal**	25 (25)	
** Musculoskeletal**	2 (2)	
**ICU length of stay median days [IQR]**	9.0 [3.8–18.9]	100
**Hospital length of stay median days [IQR]**	18 [10–28]	86
**Gram-negative pathogen detected n (%)**	32 (67%)	48
**30-day mortality n (%)**	32 (32)	100

At study inclusion, the median serum concentration of procalcitonin (PCT) was 2.6 ng/mL [IQR: 0.5–11.5; *n* = 84], and C-reactive protein (CRP) was 17 mg/dL [IQR: 11–28; *n* = 82]. Lactate was measured at a median concentration of 1.4 mmol/L [IQR: 0.9–1.9; *n* = 84]. A more in-depth characterization of the septic cohort is shown in Table 1.

The non-septic surgical control cohort comprised 18 patients, of whom 12 were male (66%), with a median age of 70 years [IQR: 56–75]. Baseline characteristics of the control cohort are provided in Supplementary Table 2.

### Validation of phospho-TLR4 assay in cell culture

The specificity of the established PLA assay was validated by downregulation of the TLR4 transcript in THP-1 macrophages (see Supplementary Materials). An average downregulation of 53% was achieved in three independent experiments, as verified by qPCR (Supplementary Fig. 1A). In immunofluorescence experiments, we observed a visibly lower protein expression of TLR4 in the cells (Supplementary Fig. 1C and D). Subsequently, TLR4 phosphorylation yielded significantly lower signals per cell in siRNA-treated cells (relative reduction in signal to 67%), than in control cells (*p* = 0.04; Supplementary Fig. 1B, E and F).

To further validate the feasibility of our phospho-TLR4 PLA assay, we tested activation dynamics in THP-1 macrophages and compared this profile with the TLR4-MyD88 protein interaction, which is expected to occur directly after the initial phosphorylation of TLR4. Our results show a very similar profile, with maximal activation at one second LPS stimulation and a subsequent deactivation at 30 s (Supplementary Fig. 3). These results strengthen the validity of our technique, which we subsequently used to measure TLR4 activation in 100 septic patients.

### TLR4 activation is overall low in septic patients

**Fig. 1:**
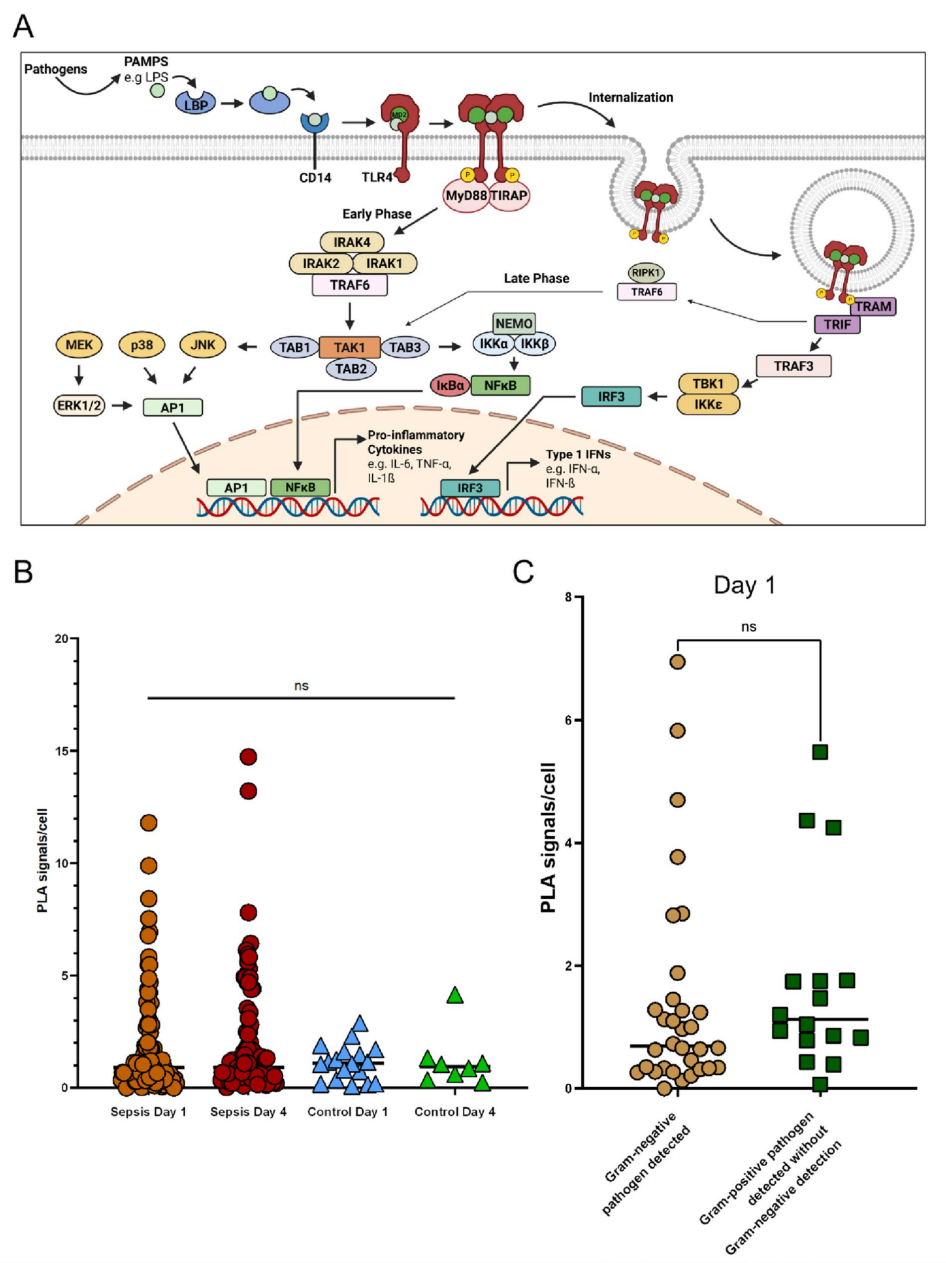
**A.** Simplified representation of TLR4 signalling upon recognition of LPS during sepsis. LPS binding to the TLR4–MD2 complex induces receptor dimerization, phosphorylation, and recruitment of MyD88/TIRAP (early phase) or TRAM/TRIF (late phase). Downstream activation of MAPKs (ERK1/2, p38, JNK), NFκB, and AP-1 promotes transcription of pro-inflammatory cytokines such as IL-6, TNF-α, and IL-1β, while TRIF-dependent activation of IRF3 induces type I interferons. Created with BioRender.com. **B.** Quantification of TLR4 PLA signals in septic patients on day 1 (orange, *n* = 100) and day 4 (red, *n* = 98), compared with non-septic surgical controls on day 1 (blue, *n* = 18) and day 4 (green, *n* = 9). **C.** Comparison of TLR4 PLA signals in samples with detected Gram-negative pathogens (yellow, *n* = 32) and Gram-positive pathogens without detection of Gram-negative pathogens (green, *n* = 16) on day 1, Mann-Whitney-U *p* = 0.172. AP-1, activator protein 1; CD14, cluster of differentiation 14; ERK1/2, extracellular signal-regulated kinase 1/2; IκBα, inhibitor of κB alpha; IKKα/IKKβ, IκB kinase α/β; IKKε, IκB kinase epsilon; IRAK1/2/4, interleukin-1 receptor-associated kinase 1/2/4; IRF3, interferon regulatory factor 3; JNK, c-Jun N-terminal kinase; LBP, lipopolysaccharide-binding protein; MAPK, mitogen-activated protein kinase; MD2, myeloid differentiation factor 2; MEK, mitogen-activated protein kinase kinase; MyD88, myeloid differentiation primary response 88; NEMO, NFκB essential modulator; NFκB, nuclear factor κB; PAMP, pathogen-associated molecular pattern; PLA, proximity ligation assay; RIPK1, receptor-interacting serine/threonine-protein kinase 1; Table 1/2/3, TAK1-binding protein 1/2/3; TAK1, transforming growth factor-β-activated kinase 1; TBK1, TANK-binding kinase 1; TIRAP, Toll/interleukin-1 receptor domain–containing adaptor protein; TLR4, Toll-like receptor 4; TRAF3/6, TNF receptor-associated factor 3/6; TRAM, TRIF-related adaptor molecule; TRIF, TIR-domain–containing adapter-inducing interferon-β

**Fig. 2:**
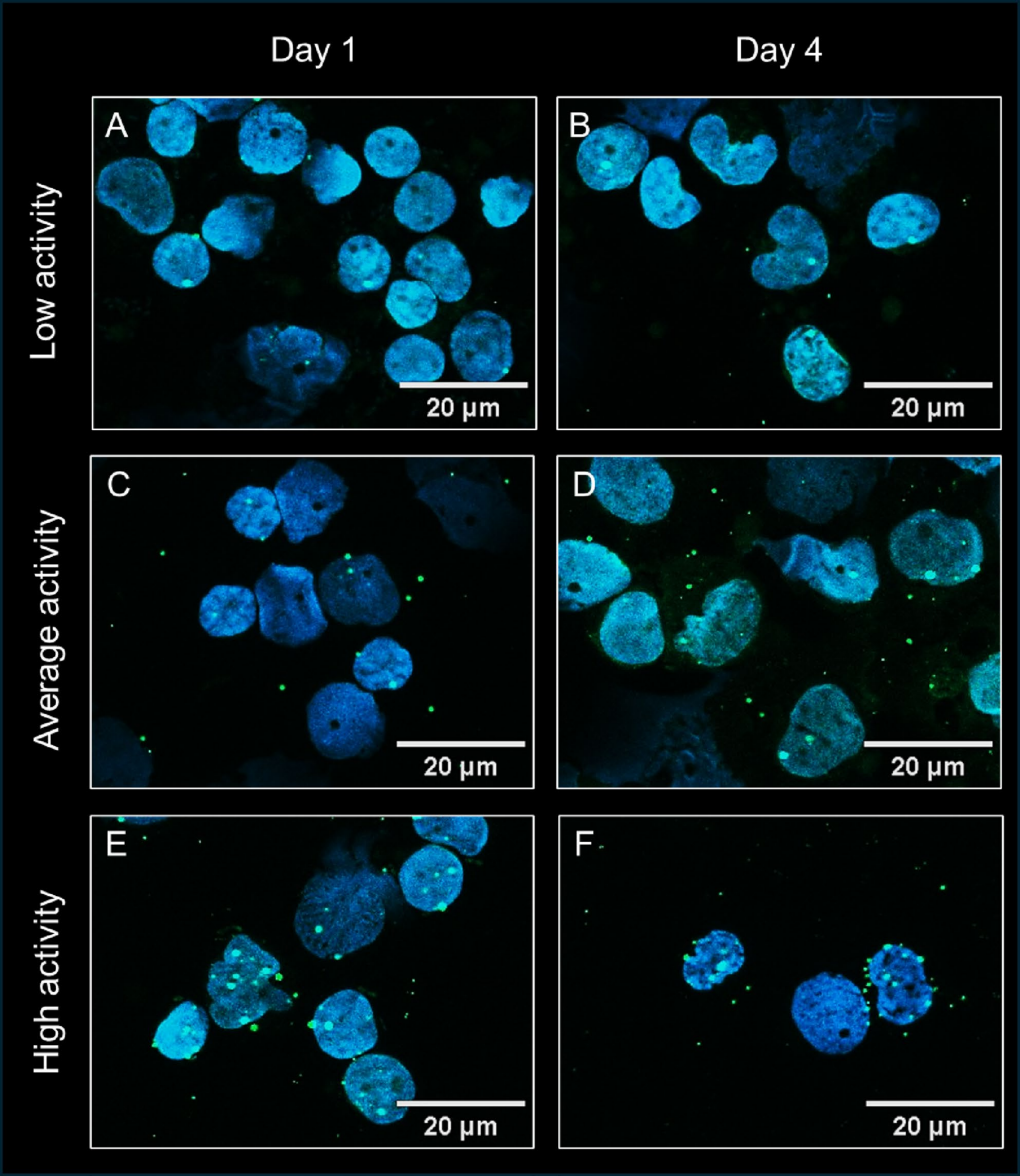
TLR4 activation in PBMCs from septic patients. Representative microscopic images illustrating low, intermediate, and high TLR4 phosphorylation signals in PBMCs from patients with sepsis at day 1 and day 4. Nuclei are shown in blue (DAPI), and PLA signals are depicted as green dots (ATTO488). Scale bar: 20 μm. (**A**) Day 1, low activity. (**B**) Day 4, low activity. (**C**) Day 1, intermediate activity. (**D**) Day 4, intermediate activity. (**E**) Day 1, high activity. (**F**) Day 4, high activity. Images were selected for illustrative purposes to represent the observed range of PLA signal densities and were not used to define formal activation categories

The TLR4 median activation at study inclusion was 0.90 [IQR: 0.42–1.92] signals per cell and 4 days later the median activation was 0.91 [IQR: 0.44–2.03]) signals per cell (Fig. 1B). Representative PLA images illustrating low, intermediate, and high levels of TLR4 activation in PBMCs at day 1 and day 4 are shown in Fig. 2.

**Fig. 3:**
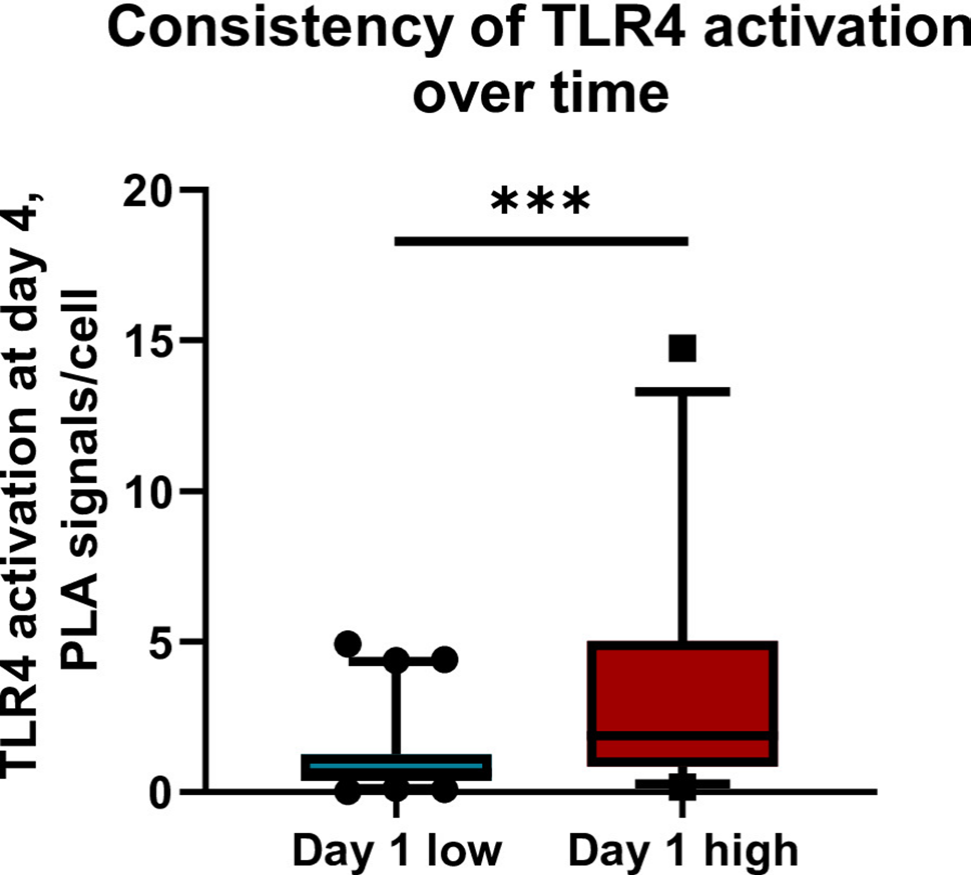
Patients were stratified into low and high TLR4 activation groups based on day 1 values. TLR4 activation at day 4 (PLA signals per cell) was compared between both groups. Patients with high TLR4 activation on day 1 showed significantly higher TLR4 activation at day 4 compared with patients with low activation on day 1. In addition, Spearman’s rank correlation analysis demonstrated a moderate positive association between TLR4 activation at day 1 and day 4 (*r* = 0.518, *p* < 0.001), indicating temporal consistency of receptor activation. Data are presented as boxplots with individual data points. Statistical significance was assessed using the Mann–Whitney U test (***p < 0.001)

Interestingly, we did not observe a significant difference in TLR4 activation when Gram-negative pathogens were detected. Of the 100 included patients, relevant bacterial pathogen classification was available for 48 patients. Of these, in 32 (67%) a Gram-negative pathogen could be detected (Table 1). The median TLR4 activation on day 1 was 0.70 signals per cell [IQR: 0.33–1.32] in patients where a Gram-negative pathogen was detected, and 1.12 signals per cell [IQR: 0.82–1.75] in patients with Gram-positive pathogen detection without Gram-negative detection (*p* = 0.172). Similar results were obtained for day 4 (1.09 [0.50–2.24] vs. 1.65 [0.82–3.75] in patients with Gram-negative pathogen detection and patients with Gram-positive pathogen detection without Gram-negative detection, respectively, *p* = 0.271; Supplementary Fig. 5A).

To assess temporal consistency of TLR4 activation, day 1 and day 4 values were compared using Spearman’s rank correlation. TLR4 activation at day 1 significantly correlated with activation at day 4 (Spearman *r* = 0.518, *p* < 0.001), indicating that patients tended to maintain their relative activation levels over time. Consistently, patients stratified as having high TLR4 activation on day 1 showed significantly higher activation levels at day 4 compared with patients with low activation on day 1 (*p* < 0.001, Mann–Whitney U test) (Fig. 3).

No significant correlations were found between TLR4 activation and cytokine levels measured on the same day. However, higher TLR4 activation on day 1 was associated with increased IFN-γ and TNF-α serum concentrations on day 4 (*p* = 0.024 and *p* = 0.047, respectively). Further data on cytokine concentrations are provided in Table 2.

**Table 2:** Concentration of cytokines at day 1 and day 4

Cytokine pg/mL	Day 1	Day 4	*p* - value
**IL-1β median [IQR]**	5.51 [2.70–8.44]	5.51 [3.37–11.1]	0.175
**IFN-α2 median [IQR]**	1.75 [1.26–2.89]	1.75 [1.17–2.29]	0.328
**IFN-γ median [IQR]**	3.85 [0.00-8.65]	4.00 [0.00-8.59]	0.367
**TNF-α median [IQR]**	6.26 [0.00-9.13]	6.69 [0.00-10.1]	0.656
**MCP1 median [IQR]**	250 [131–567]	242 [136–392]	0.208
**IL-6 median [IQR]**	181 [54.3–453]	57.6 [24.6–187]	**< 0.001**
**IL-8 median [IQR]**	77.2 [33.0-137]	48.0 [27.9–101]	**0.020**
**IL-10 median [IQR]**	6.91 [2.14–17.4]	6.54 [0.00-11.5]	0.052
**IL-12_p70 median [IQR]**	2.66 [1.82–4.39]	2.85 [1.90–3.93]	0.338
**IL-17a median [IQR]**	0.455 [0.270–0.670]	0.500 [0.270–0.840]	0.318
**IL-18 median [IQR]**	305 [119–614]	388 [143–757]	**0.019**
**IL-23 median [IQR]**	6.13 [0.00-19.8]	9.98 [0.00-26.1]	**0.037**
**IL-33 median [IQR]**	19.3 [12.0-33.1]	22.3 [13.0-45.6]	0.273

Cytokine concentration is depicted as median [IQR] in pg/mL. Statistical analysis was done using a paired Wilcoxon signed rank test. A *p* value of at least 0.05 was considered statistically significant (bold)

Overall, the cytokine dynamics indicate a shift from an acute inflammatory response, reflected by higher IL-6 and IL-8 levels, towards a more sustained and potentially reprogrammed immune state, characterized by increased levels of inflammasome-associated cytokines such as IL-18 and IL-23 (see Table 2).

### Plasma proteome is not associated with TLR4 activation

The plasma protein composition of 84 patients (35 TLR4-high, 49 TLR4-low) at day 1 was assessed. After identifying 440 protein groups we could not detect any significant changes between the two groups (Supplementary Data, Supplementary Table 2).

### Activation of TLR4 in sepsis is detrimental for 30-day survival

While the overall signal of TLR4 activation was low, we found TLR4 activation status to be associated with the 30-day survival. Kaplan-Meier analysis was performed after determining the point of best discrimination between sepsis survivors and non-survivors by ROC-analysis using the Youden index. The identified threshold was 1.11 signals per cell for day 1 and 0.65 signals per cell for day 4. As Kaplan-Meier analysis showed, patients with a TLR4 activation status that was below cut-off value, had a significantly greater chance of survival than those with a higher TLR4 activation status.

**Fig. 4:**
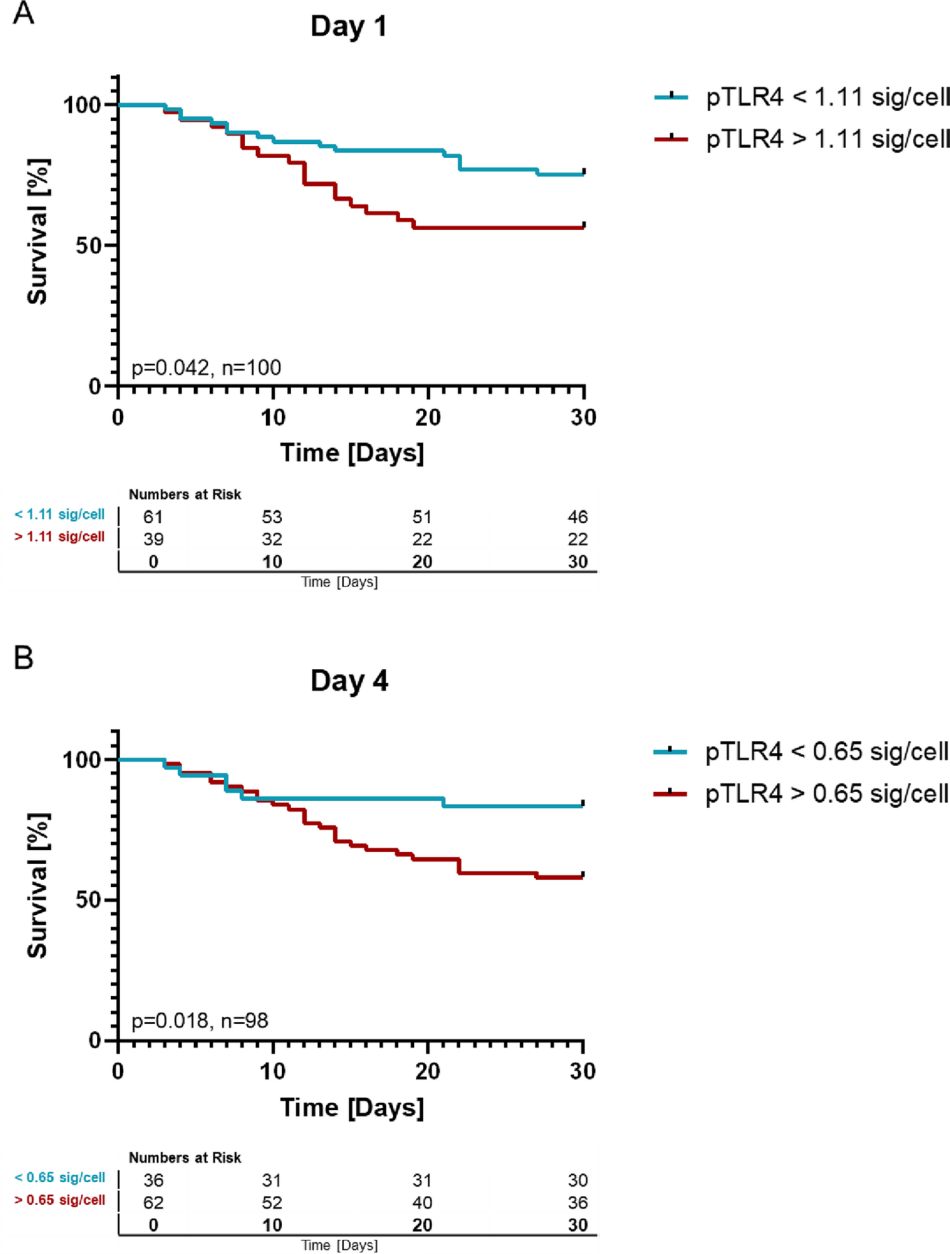
Kaplan-Meier analysis comparing 30-day survival of patients with TLR4 activation above (red) and below (blue) threshold value. **A.** Patients with a low TLR4 activation status (blue) at day 1 showed a greater chance of survival for the following 30 days than those with a high TLR4 activation status (red) (*p* = 0.041, HR: 2.028 [95%CI:1.011–4.067]). **B.** Patients with a low TLR4 activation status (blue) at day 4 also exhibited a better 30-Day survival rate than patients with elevated TLR4 activation (red) (*p* = 0.018, HR: 2.766 [95%CI: 1.138–6.726])

**Fig. 5:**
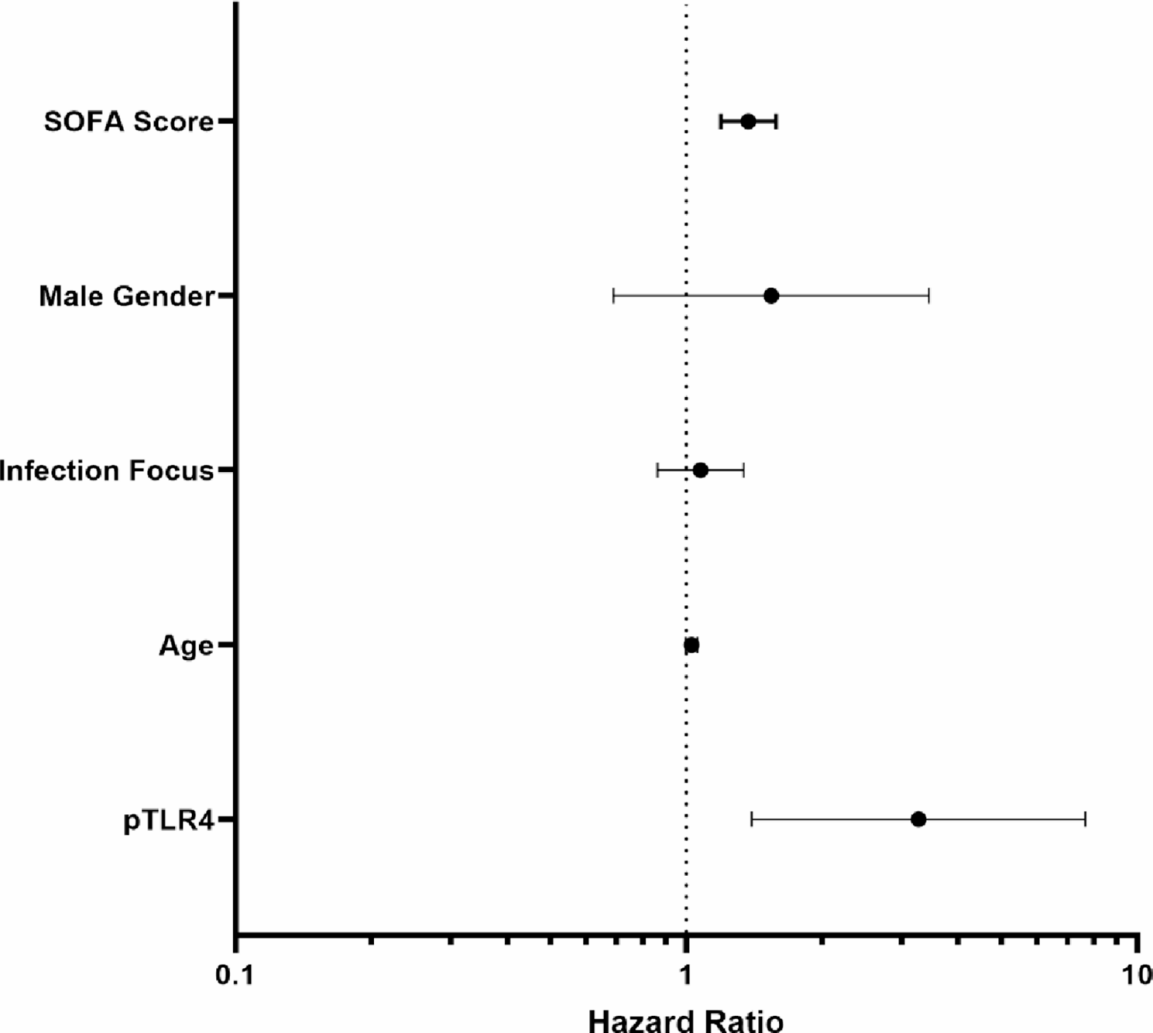
Forest plot showing hazard ratios of the multivariable Cox regression analysis. SOFA Score at study inclusion (HR: 1.374 [95%CI: 1.193–1.583], *p* < 0.001) and phospho-TLR4 threshold at day 1 (HR: 3.277 [95%CI: 1.399–7.678], *p* = 0.006) were independently associated with 30-day survival in this model. The other variables were not independently associated with survival (Age: HR: 1.03 [95%CI: 1.000-1.050], *p* = 0.080; Male Gender: HR: 1.42 [95%CI: 0.660–3.050], *p* = 0.380; Infection Focus: HR: 1.061 [95%CI: 0.790–1.190], *p* = 0.290)

At day 1, patients with high TLR4 activation showed lower 30-day survival than patients with low TLR4 activation (56.4% vs. 75.4%, *p* = 0.041, log-rank test; Fig. 4). This difference was also observed at day 4 (58.1% vs. 83.3% *p* = 0.018, high TLR4 activation status vs. low TLR4 activation status, respectively, Fig. 4). The extent of this effect is reflected in the hazard ratios, which were determined univariately to be 2.028 (95%CI: 1.011–4.067, *p* = 0.048) for day 1 and 2.766 (95%CI: 1.138–6.726, *p* = 0.025) on day 4. In a multivariable Cox regression analysis adjusted for SOFA score at admission, age, infection focus, and sex, high TLR4 activation on day 1 remained associated with 30-day mortality (HR 3.277, 95% CI 1.399–7.678, *p* = 0.006; Fig. 5).

Therefore, we found that in this cohort, elevated levels of phosphorylated TLR4 were indeed detrimental to patient survival.

### Zonulin, a marker for leaky gut syndrome, is associated with TLR4 activation

In order to assess gastrointestinal leakage of LPS into the bloodstream as a possible way to activate TLR4, we measured serum concentrations of zonulin at day 1 after sepsis diagnosis. The median zonulin concentration in serum of sepsis patients was 1.4 ng/mL. Zonulin concentration was not associated with 30-day mortality (1.38 ng/mL vs. 1.83 ng/mL survivors and non survivors respectively, *p* = 0.43). However, we could identify a weak association with TLR4 activation on day 1 (Pearson correlation *r* = 0.237, *p* = 0.046) the biological significance of this finding, however, remains to be determined (Supplementary Fig. 4).

## Discussion

In this multicentre study, we show that TLR4 activation is overall low in septic patients, but when elevated, it predicts 30-day mortality independently of SOFA score and other covariates. This subgroup of patients can be conceptualized as a TLR4-activated sepsis endotype, characterized by phosphorylation-dependent receptor activation and poor prognosis.

These observations provide a novel, functional perspective on TLR4 signalling in human sepsis, made possible through direct quantification of receptor activation using a phosphorylation-based PLA assay. Previous sepsis trials targeting TLR4 inhibition, such as the ACCESS study [18], assumed universal receptor activation, relying on surrogate markers such as IL-6 or other cytokines. Our findings challenge this assumption by providing the first direct quantification of phosphorylated TLR4 in patient-derived cells, showing that activation is present only in a subset of patients. These results redefine the clinical context in which TLR4 signalling should be considered a therapeutic target.

While our analysis focuses on circulating PBMCs, this compartment provides a clinically accessible window into systemic immune activation. However, PBMC-based TLR4 phosphorylation does not fully capture tissue-specific signaling processes, particularly within the vascular endothelium and tissue-resident immune cells, which play central roles in sepsis-associated organ dysfunction [26]. Thus, our findings should be interpreted as reflecting systemic immune-cell signaling rather than organ-specific pathophysiology. Nevertheless, circulating immune cells represent a relevant and translationally applicable source for functional biomarker assessment.

Our results suggest that TLR4 antagonists may still hold therapeutic potential, but their application should be more selective. Importantly, the interpretation of TLR4 activation in this context is inherently asymmetric. While low or absent phosphorylation at a given time point does not exclude prior receptor activation and may reflect resolution of a preceding signalling event, receptor internalization, or endotoxin tolerance, elevated TLR4 phosphorylation provides a more direct indication of ongoing receptor activity at the time of sampling.

A key consideration is the transient nature of receptor phosphorylation. TLR4 phosphorylation is known to peak rapidly following ligand exposure and decline within a short time frame [10]. Thus, a single clinical sampling time point may not fully capture the temporal dynamics of receptor activation. This may introduce temporal sampling bias, because the measured TLR4 signal depends on the timing of blood sampling within each patient’s individual inflammatory response.

However, several observations argue against a purely stochastic sampling effect as the sole explanation for the observed interindividual variability. TLR4 activation at day 1 correlated significantly with activation at day 4, indicating that patients tended to maintain their relative activation levels over time. In addition, no significant overall shift in TLR4 activation was observed between both time points. While these findings do not prove continuous receptor activation, they are consistent with sustained or recurrent high TLR4 activation in a subset of patients rather than random capture of short-lived phosphorylation peaks alone.

Taken together, these findings indicate that low TLR4 activation remains difficult to interpret in isolation, whereas elevated TLR4 phosphorylation identifies a biologically active signalling state with temporal consistency in this cohort. This distinction suggests that high TLR4 activation may identify a clinically relevant subgroup of patients with ongoing receptor activity or a broader inflammatory activation phenotype, whereas low activation is not informative with respect to prior signalling dynamics. Notably, TLR4 activation was associated with survival in time-to-event analyses, supporting its potential relevance as a biologically informative activation marker. However, its clinical utility as a predictive biomarker will require validation in independent cohorts and should not be inferred from the present exploratory analysis alone.

The general assumption that high TLR4 activation negatively impacts patient survival seems to be valid. However, as many septic patients show a low, very short-term, or absent activation of TLR4, further inhibition of this receptor may even be detrimental in these patients. It could be argued that further inhibition of already low TLR4 activation might increase susceptibility to low-grade nosocomial infections and thereby increase mortality in the lower-risk patient group. This phenomenon has also been observed in post-hoc analyses of the ACCESS trial, which revealed a potentially worse outcome with Eritoran for patients with less severe sepsis [18]. It seems plausible that even if the high TLR4 group would benefit from the treatment, the effect would be overshadowed by the overall worse outcome in the low TLR4 group. This finding underscores that a standardized treatment of all sepsis patients may be detrimental and instead patient enrichment for high TLR4 activation and precision medicine approaches are needed to advance sepsis therapy. We therefore argue that further clinical evaluation of TLR4 inhibitors remains warranted, provided that patient selection is based on actual TLR4 activation status.

Previous studies have used surrogate markers such as IL-6 expression or NFκB translocation to infer TLR4 activation (Fig. 1A). While appropriate in vitro [10], these markers are unspecific in vivo, where multiple non-linear signalling networks converge [27, 28] and different compartments contribute to the overall plasma composition [29]. Our findings confirm this: cytokine levels as well as plasma proteome showed no consistent differences between patients with high and low TLR4 activation in PBMCs. Hence, indirect readouts are insufficient to assess receptor activation in sepsis. Mechanistic biomarker assessment may refine sepsis endotyping and guide future therapeutic stratification. In this context, we observed that higher TLR4 activation at day 1 was associated with increased levels of selected cytokines, including IFN-γ and TNF-α, at day 4. Given the highly transient nature of receptor phosphorylation, a direct mechanistic link across this time interval appears unlikely. We therefore do not interpret this finding as evidence of a temporally resolved downstream signalling cascade. Instead, this association may reflect a more sustained, system-level inflammatory state, in which early receptor activation identifies patients with a broader pro-inflammatory phenotype that persists over time. At the same time, circulating cytokine levels represent an integrated systemic signal derived from multiple cellular sources and compartments, and may therefore not directly correspond to receptor-level activation in PBMCs.

Consequently, this observation should be interpreted with caution and considered exploratory, rather than indicative of a direct causal or mechanistic relationship.

Another unexpected finding was the lack of a significant difference in TLR4 activation between patients with Gram-negative pathogen detection and patients with Gram-positive pathogen detection without Gram-negative detection. Polymicrobial infections containing Gram-negative bacteria were included in the Gram-negative pathogen detection group, because the biological question concerned potential exposure to LPS. One possible explanation is sepsis-associated dysbiosis and increased intestinal permeability, which are well-described features of sepsis-associated gut barrier dysfunction [30]. Translocation of Gram-negative bacteria or their components from the gut microbiome into the bloodstream could theoretically contribute to TLR4 activation in patients without detected Gram-negative pathogens. In line with this hypothesis, we observed a weak albeit significant correlation between zonulin, a marker of intestinal permeability, and TLR4 activation. However, given the low correlation coefficient and the limited sample size of this exploratory subgroup analysis, this association should be interpreted with caution and does not allow firm conclusions regarding a causal relationship. Rather, it is compatible with, but does not establish, a contribution of intestinal barrier dysfunction to systemic TLR4 activation in sepsis. Further studies are required to clarify the mechanistic relevance of this observation.

The generally low activation of TLR4 may also reflect endotoxin tolerance, which describes a reduced responsiveness to repeated LPS exposure. The phosphorylation, and hence activation, of TLR4 could represent a regulatory bottleneck contributing to this phenomenon [31]. Endotoxin tolerance may protect the body from excessive inflammation [32] but also hampers a uniform therapeutic approach. Consequently, our data argue against a one-size-fits-all approach to TLR4 inhibition in sepsis and instead call for precision medicine guided by endotype-specific activation patterns.

### Limitations

This study has several limitations. Although based on a multicentric, prospective cohort, all participating hospitals were located within a single geographical region in Germany, which may limit generalisability to other populations. Functional validation of phosphorylated TLR4 was performed in vitro using established gold-standard methods but not in patient-derived cells beyond PLA-based quantification. External validation in independent sepsis cohorts will therefore be necessary to confirm our findings. Furthermore, as an observational study, this work cannot establish causality between TLR4 activation and mortality, and residual confounding cannot be excluded. In addition, PBMC isolation, cryopreservation, and thawing may affect absolute receptor phosphorylation levels, although all sepsis and control samples were processed under identical conditions.

Pathogen-based subgroup analyses are limited by incomplete microbiological pathogen identification. Although blood cultures were obtained systematically and complemented by clinically relevant non-blood microbiological findings where available, bacterial pathogen classification remained possible only in a subset of patients. Moreover, classification as Gram-negative pathogen detection does not imply exclusively Gram-negative infection, as polymicrobial infections containing Gram-negative bacteria were included in this group. Therefore, analyses according to pathogen detection status should be considered exploratory and underpowered.

In addition, the transient nature of receptor phosphorylation and the use of single time point measurements may limit the ability to fully capture the temporal dynamics of TLR4 activation. Consequently, low TLR4 activation at a given time point cannot be interpreted as absence of prior receptor activation, but may reflect resolution of a preceding activation peak or the presence of endotoxin tolerance. This introduces a potential temporal sampling bias that should be considered when interpreting the results.

Accordingly, the interpretation of TLR4 activation, particularly in the context of low activation states, remains exploratory and requires prospective validation, especially with regard to biomarker-guided therapeutic stratification.

## Conclusion

In conclusion, our findings suggest that TLR4 inhibition should not be dismissed but applied selectively to patients with demonstrable receptor activation. The marked heterogeneity of TLR4 signalling among septic patients highlights the need for biomarker-guided therapeutic strategies. Prospective, enrichment-based clinical trials assessing TLR4 activation status prior to intervention will be essential to determine whether targeted inhibition can improve outcomes. Ultimately, individualized modulation of innate immune networks may represent a key step toward precision medicine in sepsis.

## Supplementary Information

Below is the link to the electronic supplementary material.

Supplementary Material 1.

Supplementary Material 2.

Supplementary Material 3.

Supplementary Material 4.

Supplementary Material 5.

Supplementary Material 6.

## Data Availability

Deidentified individual participant data underlying the conclusions of this study, together with the corresponding data dictionary, will be available from the time of publication without a predefined end date upon reasonable written request, including a study plan, to the corresponding author. No participant data containing direct identifiers will be shared. Access to the data will be granted to qualified researchers for scientific, non-commercial purposes after evaluation of the request and approval by the corresponding author, and data will be shared under a data access agreement where appropriate. Related study documents registered at the German Clinical Trials Register (DRKS00018871) that are not publicly available will not be shared.The proteomics data generated in this study have been deposited in the PRIDE Archive under accession number PXD055932 (https://www.ebi.ac.uk/pride/archive/projects/PXD055932).

## Abbreviations

ACCESS: A Controlled Comparison of Eritoran and Placebo in Patients with Severe Sepsis
AP-1: Activator protein 1
CD14: Cluster of differentiation 14
CRP: C reactive protein
DAMPs: Damage associated molecular patterns
DAPI: 4′,6 diamidino 2 phenylindole
DRKS: Deutsches Register Klinischer Studien (German Clinical Trials Register)
EDTA: Ethylenediaminetetraacetic acid
ERK1/2: Extracellular signal regulated kinase 1/2
IFN-γ: Interferon gamma
IKKα/IKKβ: IκB kinase alpha/beta
IKKε: IκB kinase epsilon
IκBα: Inhibitor of κB alpha
IL-6: Interleukin 6
IRAK1/2/4: Interleukin 1 receptor associated kinase 1/2/4
IRF3: Interferon regulatory factor 3
IQR: Interquartile range
JNK: c Jun N terminal kinase
LBP: Lipopolysaccharide binding protein
LOD: Limit of detection
LPS: Lipopolysaccharide
MAPK: Mitogen activated protein kinase
MD2: Myeloid differentiation factor 2
MEK: Mitogen activated protein kinase kinase
MyD88: Myeloid differentiation primary response 88
NEMO: NFκB essential modulator
NF-κB: Nuclear factor kappa B
PAMP: Pathogen associated molecular pattern
PBMCs: Peripheral blood mononuclear cells
PBS: Phosphate buffered saline
PCT: Procalcitonin
PLA: Proximity ligation assay
PRIDE: Proteomics Identifications Database
PRRs: Pattern recognition receptors
qPCR: Quantitative polymerase chain reaction
RIPK1: Receptor interacting serine/threonine protein kinase 1
ROC: Receiver operating characteristic
ROS: Reactive oxygen species
SEPSIS-3: Third International Consensus Definitions for Sepsis and Septic Shock
SOFA: Sequential Organ Failure Assessment
Table 1/2/3: TAK1 binding protein 1/2/3
TAK1: Transforming growth factor beta activated kinase 1
TBK1: TANK binding kinase 1
TIRAP: Toll/interleukin 1 receptor domain containing adaptor protein
TLR: Toll like receptor
TLR4: Toll like receptor 4
TRAF3/6: TNF receptor associated factor 3/6
TRAM: TRIF related adaptor molecule
TRIF: TIR domain containing adapter inducing interferon beta
TNF-α: Tumor necrosis factor alpha
