# Supplementary material for: Phosphorylated toll-like receptor 4 defines a high-risk sepsis endotype
Source: Crit Care. 2026 May 30;30:285. doi: 10.1186/s13054-026-06115-5 (PMC13227810; doi:10.1186/s13054-026-06115-5)
Supplement: Supplementary file 1 — Supplementary Material 1. [file 13054_2026_6115_MOESM1_ESM.docx]

**Supplementary Data**

**Methods:**

*RNA interference*

Cells were transiently transfected with a TLR4 Silencer™ Select Validated siRNA (Ambion, Carlsbad, USA) directed against TLR4. Briefly, in 6-well plates (Sarstedt) 0.5*10^6^ THP-1 cells (DSMZ, Braunschweig, Germany) were seeded and treated with 50 ng/mL Phorbol-12-myristate-13-acetate (PMA) (Sigma-Aldrich, St. Louis, USA) in RPMI-1640 medium with 1 % (v/v) Gibco™ penicillin/ streptomycin (P/S) (Thermo Fisher Scientific, Waltham, USA) for 24h.

After a resting period of 72h in RPMI-1640 medium (Sigma-Aldrich, St. Louis, USA supplemented with 10 % (v/v) Fetal Bovine Serum Supreme (FBS) (PAN-Biotech, Aidenbach, Germany) and 1 % P/S, transfections were performed using Lipofectamine™ RNAiMAX (Invitrogen, Carlsbad, USA) following the manufacturer's instructions. Cells were transfected with 2.5 pmol siRNA using 1.5 μl transfection reagent. After 48 h of transfection TLR4 gene expression was measured by quantitative reverse transcription PCR (qRT-PCR).

*Quantitative Reverse Transcription Polymerase Chain Reaction*

After cells were transfected, RNA was isolated using the RNeasy Kit (Qiagen) according to manufacturer’s instructions. RNA concentration was measured using a NanoDrop One (Thermo Fisher Scientific, Waltham, USA) and 1 μg of total RNA was reverse transcribed using the High Capacity cDNA reverse transcription kit from Applied Biosystems (Waltham, USA) according to the manual. Using specific primers for TLR4 mRNA (Supplementary Table 1) and ß-Actin for normalization, we detected the residual expression using the GoTaq qPCR MasterMix (Promega, Madison, USA) according to manufacturer’s instructions. Following the primary qPCR protocol, a melting curve protocol was conducted in order to verify specificity of the results.

*Immunofluorescence*

In order to show a reduction of TLR4 expression at the protein level, we conducted an immunofluorescence protocol upon siRNA knockdown of the protein. For this, cells were fixed 48h after transfection using 4% formaldehyde solution in PBS at 4°C for 30 min. Cells were permeabilized using 0.1% Triton-X for 5 mins and subsequently washed with TBST. After blocking using Intercept Blocking Buffer (Li-Cor Biosciences GmbH, Bad Homburg vor der Höhe, Germany) for 30 min, the primary antibody against TLR4 (sc-293072, Santa Cruz Biotechnology, Dallas, USA), diluted 1:1000 in PBS, was incubated at 4 °C overnight. After extensive washing, secondary antibodies (Jackson ImmunoResearch, Ely, UK) were incubated for 1 h at 37 °C. Cells were counterstained with DAPI and imaged using an inverted microscope (IX51, Olympus, Tokyo, Japan).

*Antibody specificity and control experiments*

Detection of TLR4 phosphorylation in the PLA assay required the combined use of two primary antibodies directed against TLR4 (sc-293072, Santa Cruz Biotechnology, Dallas, USA) and phospho-tyrosine (p-Tyr-1000, #8954, Cell Signaling Technology, Danvers, MA, USA). To assess antibody specificity and potential unspecific binding, control experiments were performed in which primary antibodies were applied individually or in combination.

THP-1 cells were stimulated with LPS (10 µg/ml, Sigma) and incubated with rabbit anti-human phospho-tyrosine and mouse anti-human TLR4 antibodies, either alone or together under identical PLA conditions.

*Human Zonulin ELISA*

Serum concentrations of zonulin were measured using a commercially available enzyme-linked immunosorbent assay (ELISA) kit (Zonulin ELISA Kit, antibodies-online, ABIN6962693, Aachen, Germany), according to the manufacturer’s instructions. The reported detection range of the assay is 0.78–50 ng/mL, with a sensitivity of approximately 0.625 ng/mL.

Briefly, 100 µL of either standards, samples, or blank were added to a pre-coated 96-well plate and incubated for 90 minutes at 37 °C. Following removal of the liquid, 100 µL of biotinylated detection antibody (diluted 1:100 in assay buffer) was added to each well and incubated for an additional 60 min at 37 °C. Wells were then washed three times with the supplied wash buffer, followed by the addition of 100 µL of HRP-conjugated secondary antibody (also diluted 1:100), and incubated for 30 min at 37 °C. After five subsequent washing steps, 90 µL of TMB substrate solution was added and the plate was incubated in the dark for 15 min at 37 °C. The reaction was stopped by adding 50 µL of stop solution, and absorbance was immediately measured at 450 nm using a microplate reader (CLARIOstar^PLUS^, BMG LABTECH, Germany).

All reagents were equilibrated to room temperature prior to use, and standards were prepared by serial dilution as provided by the manufacturer. All measurements were performed in duplicate. Sample concentrations were calculated based on a standard curve using a four-parameter logistic (4-PL) regression model. Negative (blank) and positive controls were included on each plate to ensure assay validity.

*Plasma Proteomics*

Plasma samples were processed and analyzed as described previously Palmowski et al. (EBioMedicine, 2025), using a modified single-pot solid-phase-enhanced sample preparation (SP3) protocol.

In brief, 1 µL plasma per sample were purified using paramagnetic carboxylated beads (Cytiva Sera-Mag, GE Healthcare, Chicago, IL) and subjected to overnight digestion with trypsin (SERVA Electrophoresis, Heidelberg, Germany). For each sample, 300 nanograms of the resulting tryptic peptides were analyzed by nano-liquid chromatography coupled online to a high-resolution mass spectrometer. Measurements were done in batches using an Ultimate 3000 RSLCnano HPLC coupled to an Orbitrap Fusion Lumos or either an Ultimate 3000 or a Vanquish Neo UHPLC coupled to an Orbitrap Exploris 240 (all Thermo Scientific, Bremen, Germany). All instruments were operated in data-independent acquisition (DIA) mode. Spectral libraries were generated using FragPipe (version 17.1), and protein quantification was performed with DIA-NN (version 1.8.1), referencing the UniProt/Swiss-Prot database (release 2022_05), restricted to Homo sapiens and comprising 20,386 protein entries. Cross-batch normalization was performed using an in-house developed linear regression-based adjustment as described previously by Unterberg et al. (Crit Care, 2023). Differences between the experimental groups were assessed by t-test (unpaired, two-sided, unequal variances) corrected for multiple testing according to Benjamini-Hochberg (see Supplementary Table 2).

**Results:**

*Validation of phospho-TLR4 Assay in cell culture*

The specificity of the established PLA assay was validated by downregulating the TLR4 transcript in THP-1 macrophages. An average downregulation of 53 % was achieved in three independent experiments, as verified by qPCR (Supplementary Figure 1A). In immunofluorescence experiments, we could observe a visibly lower protein expression of TLR4 in the cells (Supplementary Figure 1C and D). Subsequently, TLR4 phosphorylation yielded significantly lower signals per cell in siRNA treated cells (relative reduction in signal to 67 %), than in control cells (p=0.04; Supplementary Figure 1B, E and F).

To further assess assay specificity and potential unspecific binding, control experiments were performed in LPS-stimulated THP-1 cells using mouse monoclonal anti-human TLR4 and rabbit monoclonal anti-phospho-tyrosine, applied either individually or in combination. Minimal PLA signal was observed when either antibody was applied alone, whereas distinct signals were detected only upon combined application, supporting proximity-dependent signal generation (Supplementary Figure 2).

To further validate the feasibility of our phosphor-TLR4 PLA assay we tested activation dynamics in THP-1 macrophages and compared this profile to the TLR4-MyD88 protein interaction, which should occur directly after the initial phosphorylation of TLR4. Our results show a very similar profile, with maximal activation at 1 s LPS stimulation and a subsequent deactivation at 30 s (Supplementary Figure 3). These results strengthen the validity of our technique, which we subsequently used to measure TLR4 activation in 100 septic patients.

*Zonulin, a marker for leaky gut syndrome, is associated with TLR4 activation*

In order to assess gastrointestinal leakage of LPS into the bloodstream as a possible way to activate TLR4, we measured serum zonulin concentration at day 1 after sepsis diagnosis. The median concentration of zonulin in the serum of sepsis patients was 1.4 ng/mL. Zonulin concentration was not associated with 30-day mortality (1.38 ng/mL vs 1.83 ng/mL survivors and non survivors respectively, p = 0.43). However, we could identify a weak association with TLR4 activation on day 1 (Pearson correlation r = 0.237, p = 0.046) the biological significance however remains to be determined (Supplementary Figure 4).

*ROC analysis of TLR4 activation at day 1 and day 4*

Receiver operating characteristic (ROC) analysis demonstrated discriminatory performance of TLR4 activation at day 1 for the prediction of 30-day mortality (AUC = 0.603, 95% CI 0.483–0.722, p = 0.093). Sensitivity was 53.1% (95% CI 36.4–69.1%) and specificity was 67.6% (95% CI 55.8–77.6%). The corresponding positive predictive value (PPV) was 43.6% (95% CI 27.8–60.4%), and the negative predictive value (NPV) was 75.4% (95% CI 62.7–85.5%).

ROC analysis demonstrated discriminatory performance of TLR4 activation at day 4 for prediction of 30-day mortality (AUC = 0.647, 95% CI 0.533–0.760, p = 0.011). Sensitivity was 81.3% (95% CI 64.7–91.1%) and specificity was 45.5% (95% CI 34.3–57.2%). The corresponding positive predictive value (PPV) was 41.9% (95% CI 29.5–55.2%), and the negative predictive value (NPV) was 83.3% (95% CI 67.2–93.6%).

**Supplementary Table 1**: Primer sequences for qRT-PCR.

| Number | Primer | Sequence | Manufacturer |
| --- | --- | --- | --- |
| 1 | TLR-4_fwd | TGCGTGGAGGTGTGAAAT | IDT |
| 2 | TLR-4_rev | CACAGCCACCAGCTTCTGT | IDT |
| 3 | Act-b_fwd | CCTTCCTGGGCATGGAGT | IDT |
| 4 | Act-b_rev | CAGGGCAGTGATCTCCTTCT | IDT |

**Supplementary Table 2**: Baseline characteristics of the control cohort. * Patients not admitted to an ICU were admitted to an intermediate care unit (IMC).

|  | **Entire cohort** | **n** |
| --- | --- | --- |
| **n** | 18 | 18 |
| **Male gender n (%)** | 12 (66) | 18 |
| **Age years median [IQR]** | 70 [56-75] | 18 |
| **SOFA score median [IQR]** | 1.5 [0-5] | 18 |
| **SAPS-2 DRG median [IQR]** | 23.5[0-29] | 18 |
| **Comorbidities n (%)** | 17 | 17 |
| **Alcohol** | 3 (18) |  |
| **Chronic kidney disease** | 2 (12) |  |
| **Hypertension** | 7 (41) |  |
| **Diabetes** | 1 (6) |  |
| **Obesity** | 2 (12) |  |
| **Cardiovascular** | 5 (29) |  |
| **Malignancies** | 15 (88) |  |
| **Nicotine** | 3 (18) |  |
| **Dialysis** | 0 (0) |  |
| **Transplantation** | 0 (0) |  |
| **COPD** | 1 (6) |  |
| **Other (lungs)** | 2 (12) |  |
| **Postoperative ICU admission n (%)*** | 11 (61) | 18 |
| **ICU length of stay median days [IQR]** | 3 [2-4] | 11 |
| **Hospital length of stay median days [IQR]** | 13 [7-22] | 18 |
| **30-day mortality n (%)** | 0 (0) | 18 |


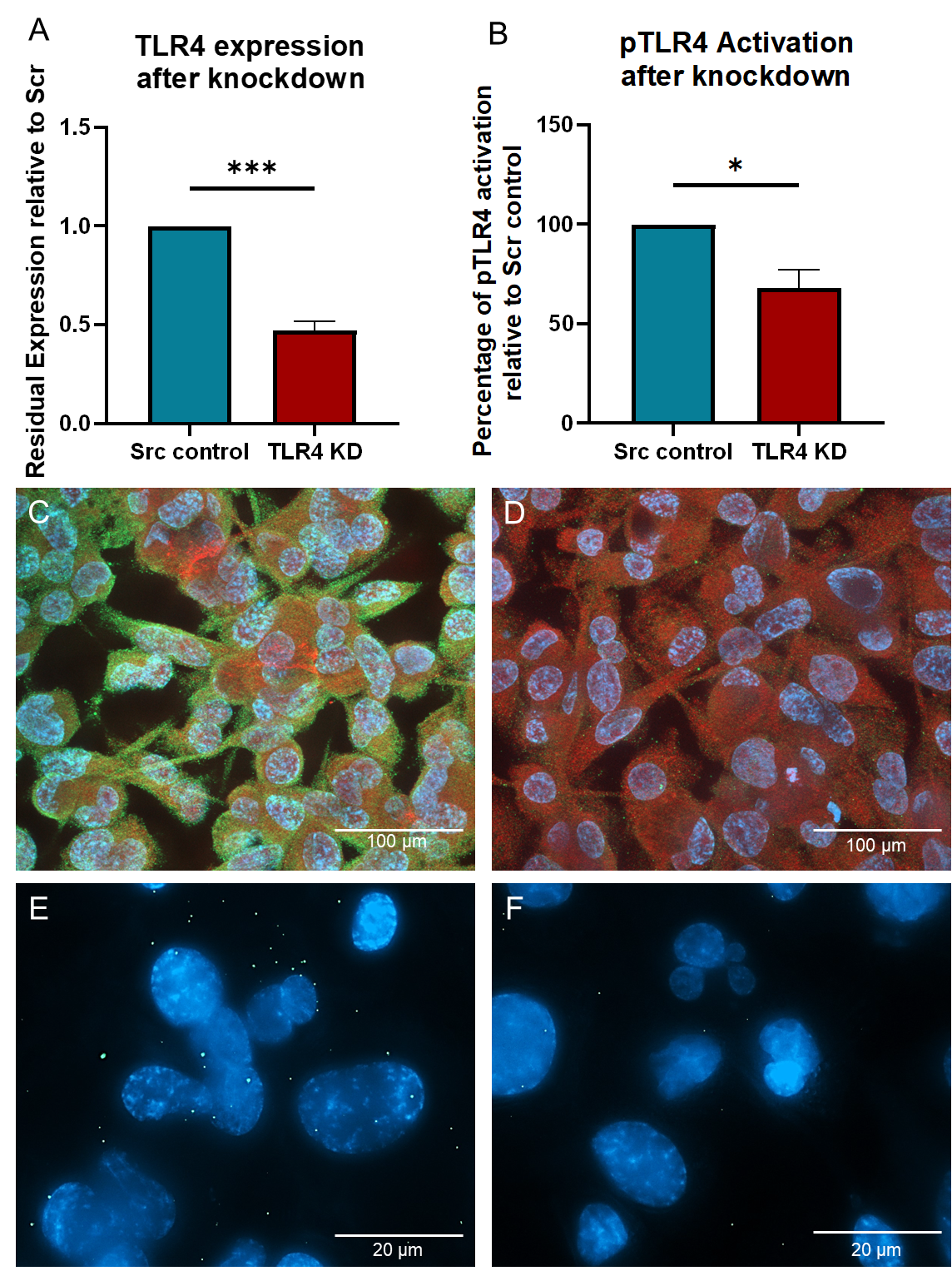


**Supplementary Figure 1:** **A.** Residual expression of TLR4 mRNA upon knockdown of TLR4. A downregulation of TLR4 to 47 % residual expression could be achieved in 3 independent experiments (p=0.007). **B.** Following the TLR4 knockdown we could show a significantly lower activation of TLR4 down to 67 % compared to Scr control (p=0.04). **C-D.** Example images of TLR4 immunofluorescence to confirm knockdown, Nuclei are depicted in blue (DAPI), TLR4 protein expression is shown in green (AF^TM^488) and phalloidin (AF^TM^594) is shown in red. **C.** src control. **D.** TLR4 knockdown. **E-F.** Example images of the activation of TLR4 (PLA). PLA signals are depicted as green dots (ATTO488) and nuclei are shown in blue (DAPI). **E.** Src control. **F.** TLR4 knockdown.

**
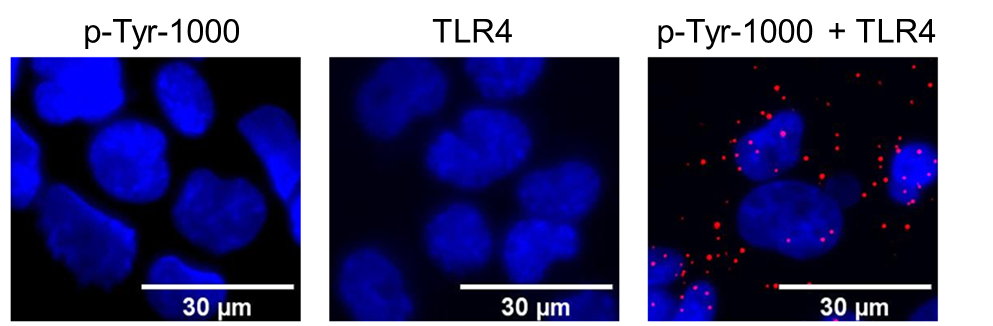
**

**Supplementary Figure 2:** Stimulated THP-1 cells were incubated with rabbit anti-phospho-tyrosine (pTyr-1000, #8954, Cell Signaling Technology, Inc., Danvers, MA, USA) and mouse anti-human TLR4 (sc-293072, Santa Cruz Biotechnology, Inc., Dallas, TX, USA), either individually or in combination under PLA conditions (see Methods). PLA signals are depicted as red dots (ATTO550) and nuclei are shown in blue (DAPI). Scale bar 30 µm.

*
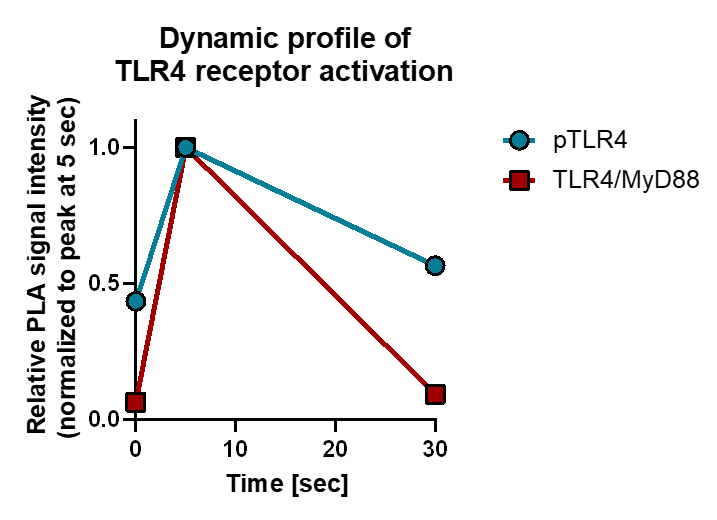
***Supplementary Figure 3:** TLR4 phosphorylation (blue) shows the same dynamic profile in THP-1 macrophages as the TLR4 / MyD88 interaction (red) that directly follows TLR4 activation.

**Supplementary Figure 4:** Serum Zonulin concentration on day one after sepsis diagnosis. Zonulin levels demonstrated a weak correlation with TLR4 activation (pTLR4) (Pearson r=0.237, p=0.046).


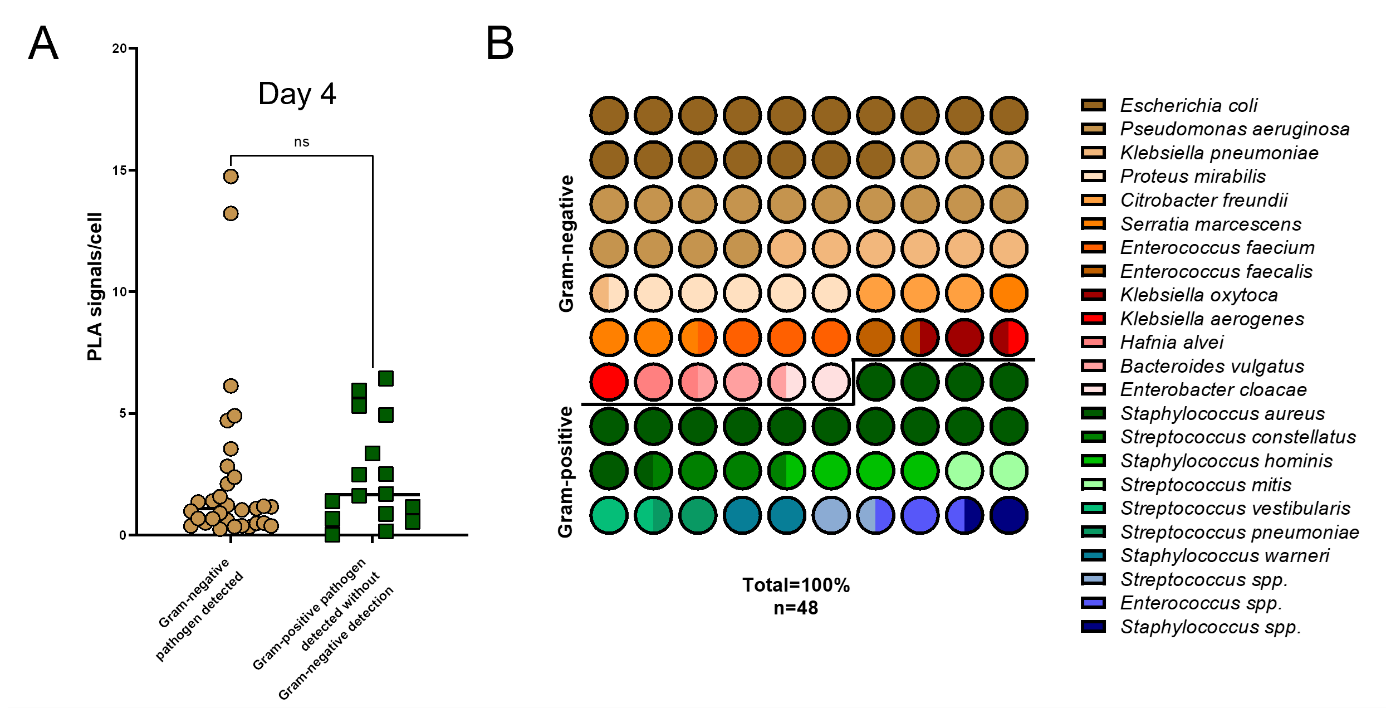


**Supplementary Figure 5:** **A.** Comparison of TLR4 PLA signals on day 4 between sepsis patients with detected Gram-negative pathogens (yellow, n = 31) and those with exclusively Gram-positive pathogen detection (green, n = 16), Mann-Whitney U p=0.271. **B.** Distribution of bacterial pathogens within the sepsis cohort, illustrating the relative contribution of Gram-negative and Gram-positive infections based on available microbiological data (n = 48).
