# Supplementary figures and images for: Phosphorylated toll-like receptor 4 defines a high-risk sepsis endotype
Source: Crit Care. 2026 May 30;30:285. doi: 10.1186/s13054-026-06115-5 (PMC13227810; doi:10.1186/s13054-026-06115-5)

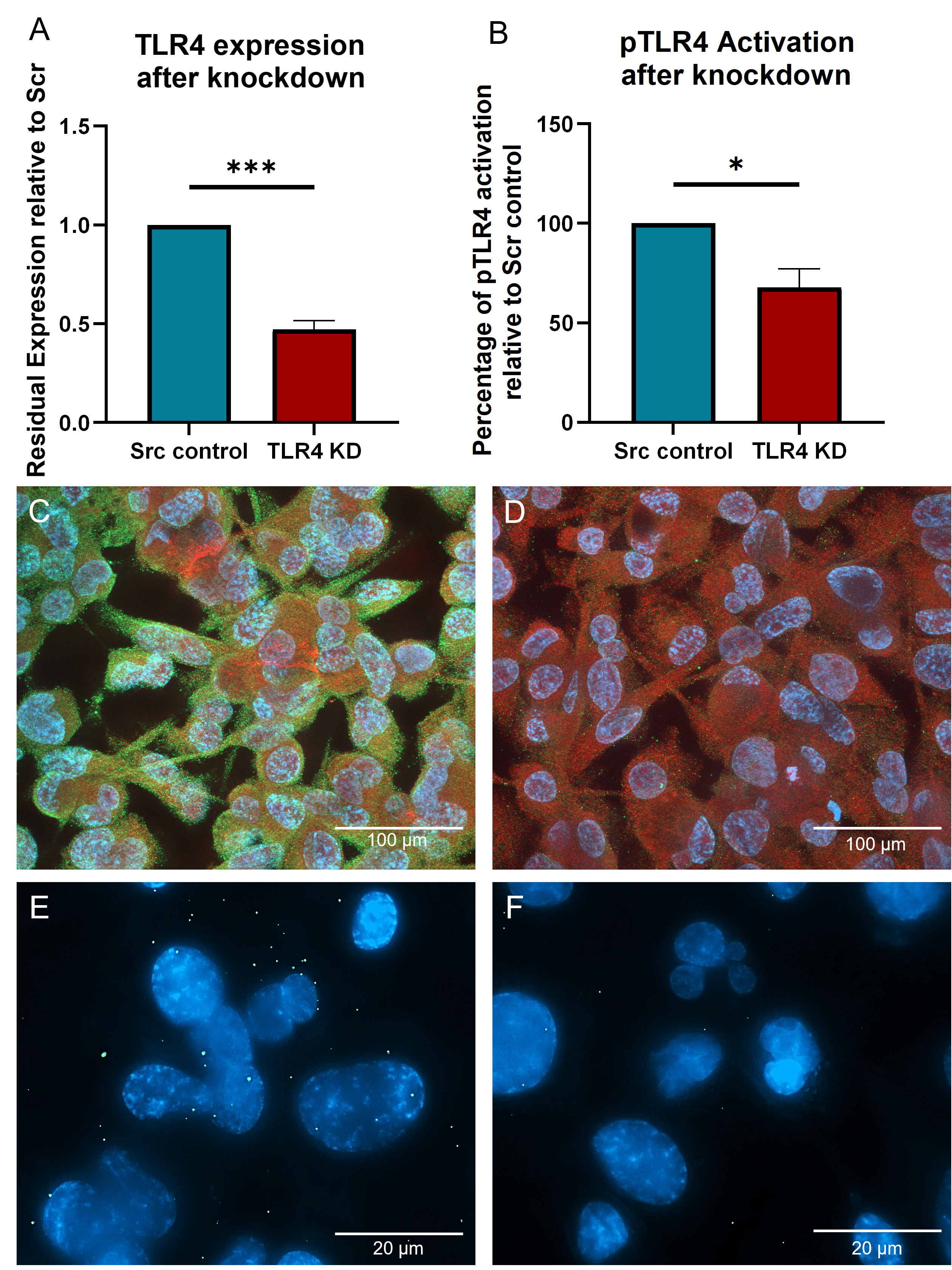

Supplement: Supplementary file 2 — Supplementary Material 2. [file 13054_2026_6115_MOESM2_ESM.png]

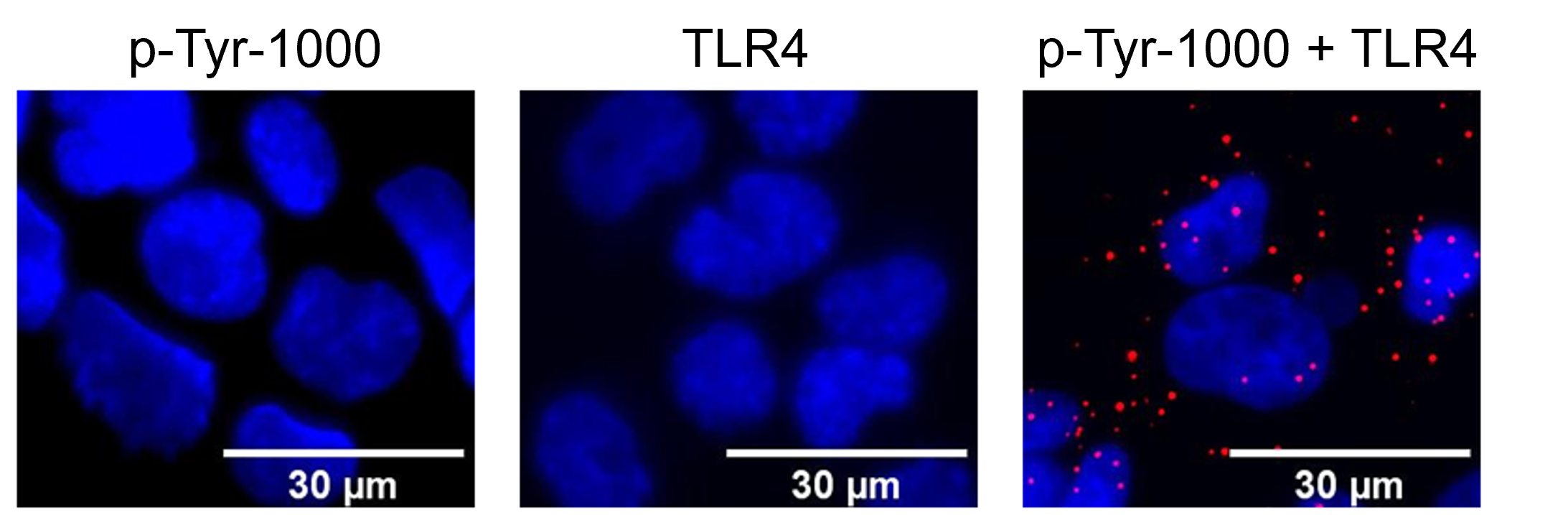

Supplement: Supplementary file 3 — Supplementary Material 3. [file 13054_2026_6115_MOESM3_ESM.png]

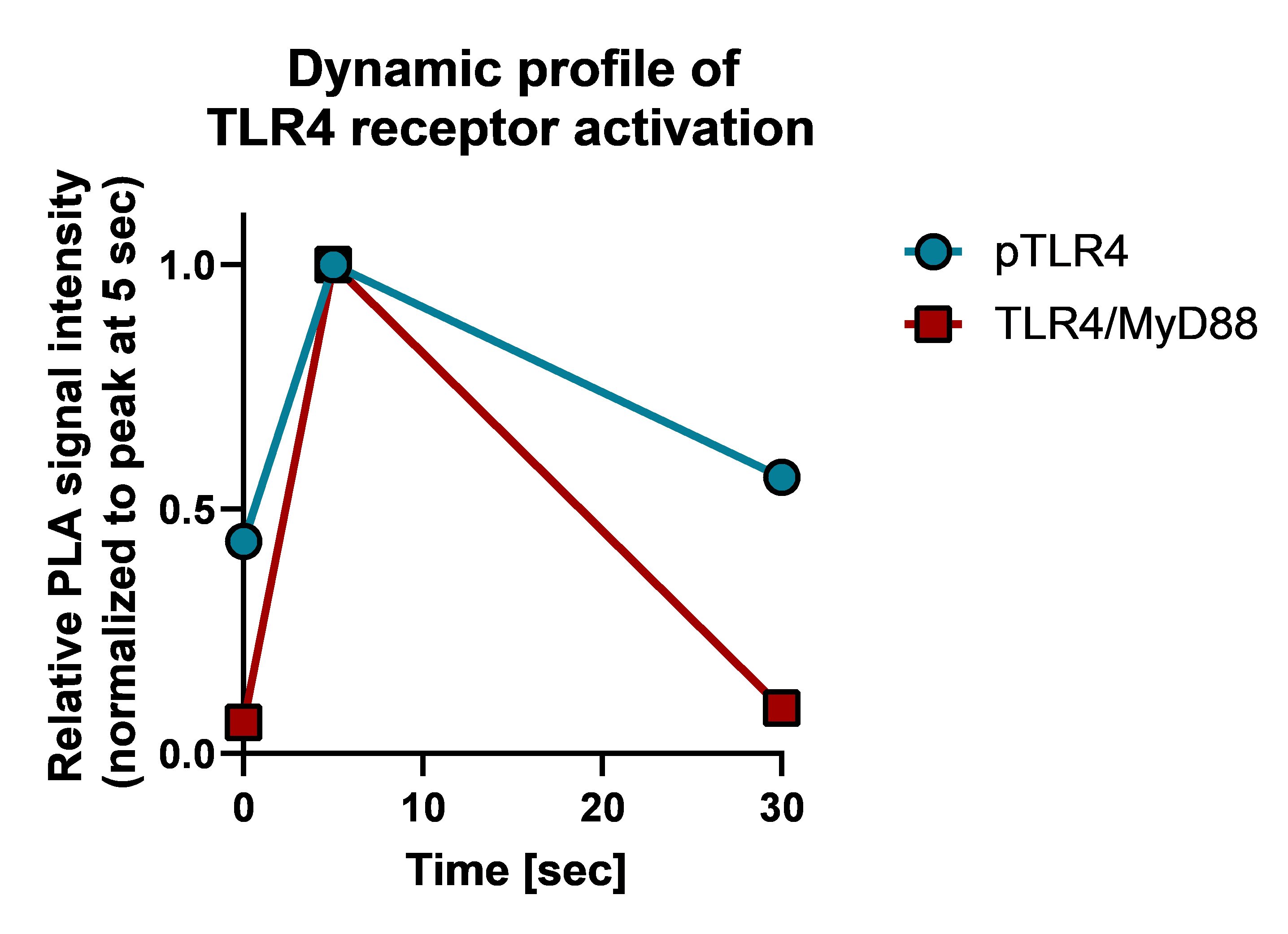

Supplement: Supplementary file 4 — Supplementary Material 4. [file 13054_2026_6115_MOESM4_ESM.png]

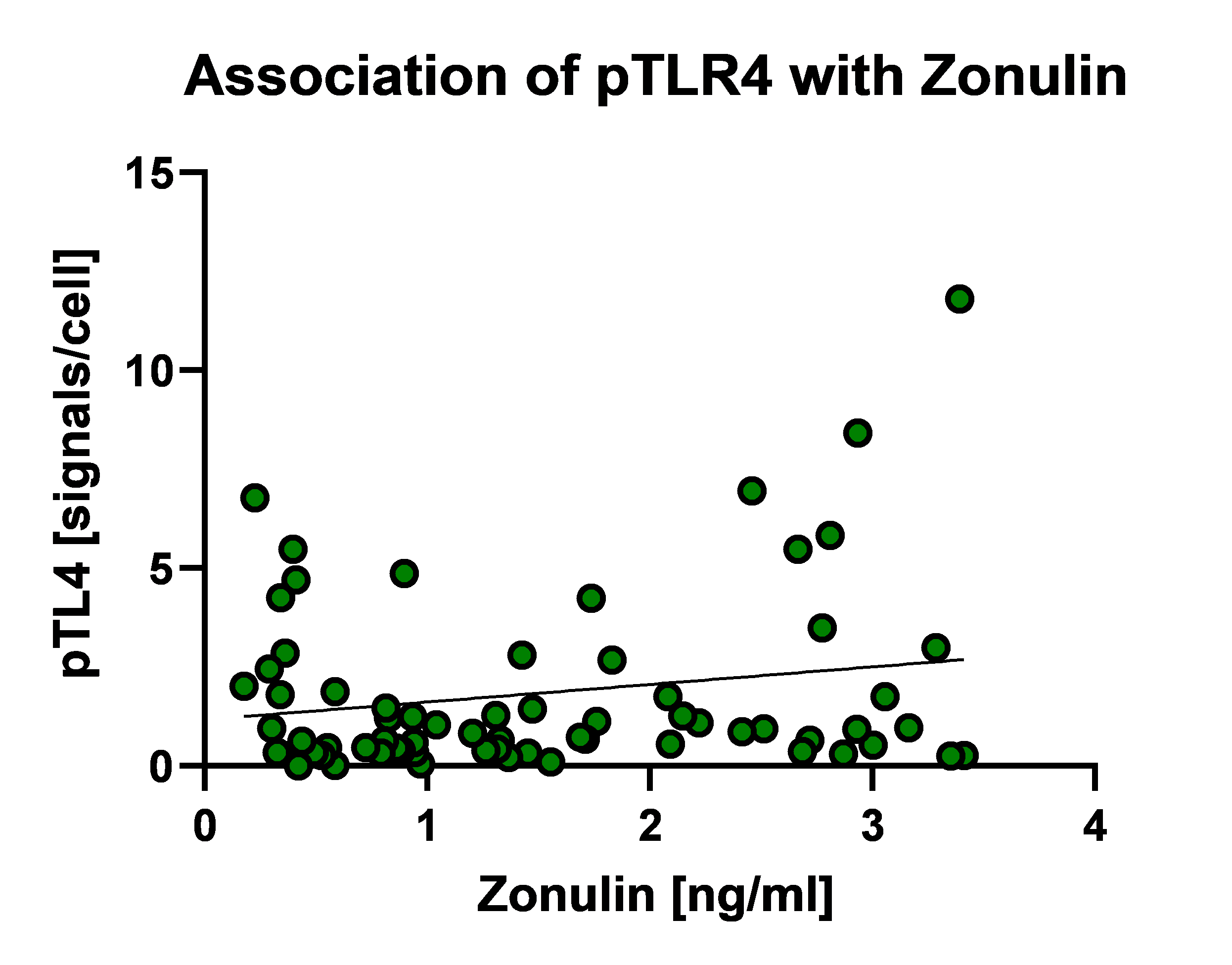

Supplement: Supplementary file 5 — Supplementary Material 5. [file 13054_2026_6115_MOESM5_ESM.png]

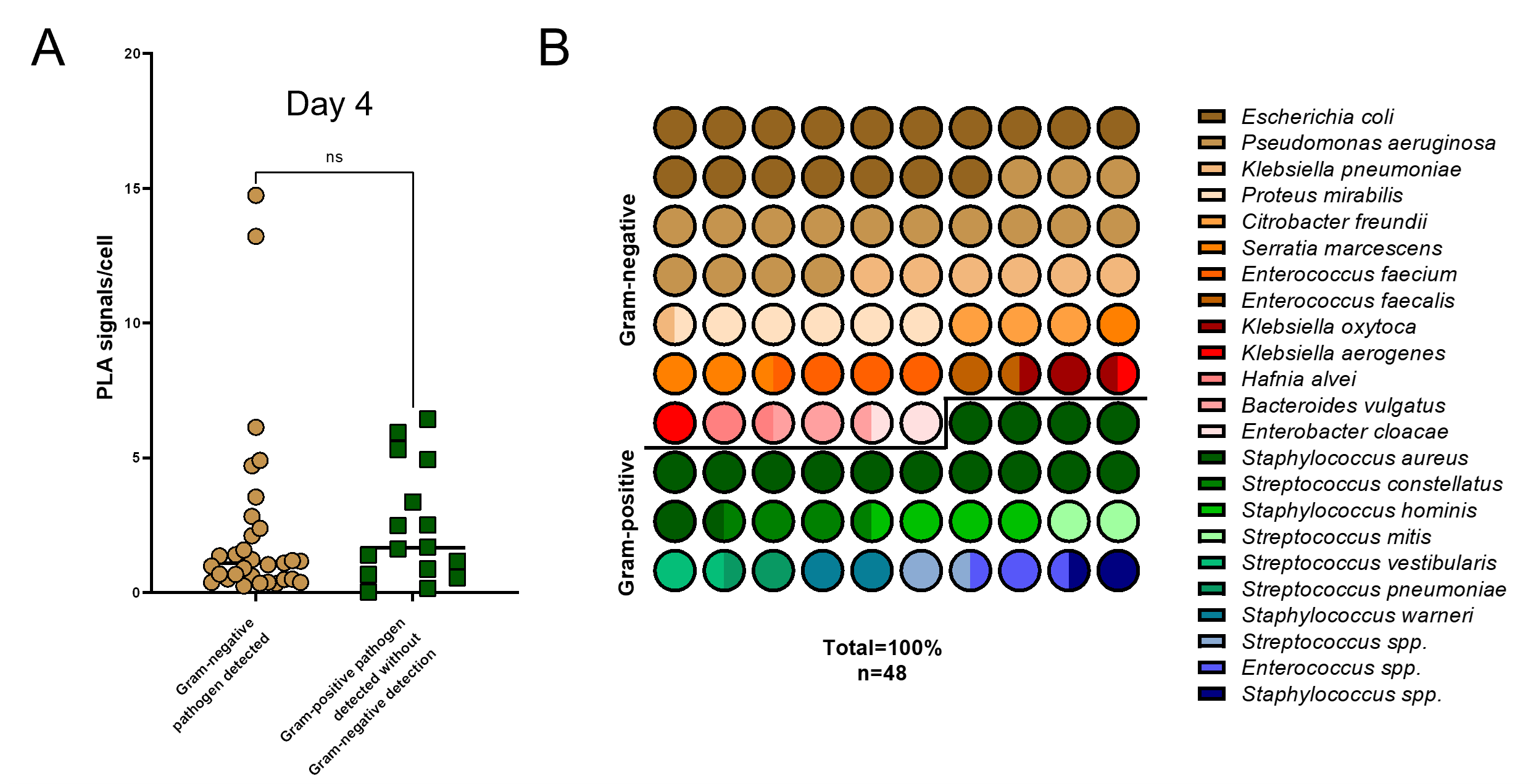

Supplement: Supplementary file 6 — Supplementary Material 6. [file 13054_2026_6115_MOESM6_ESM.png]
